# Bioprinting and In Vitro Characterization of an Eggwhite-Based Cell-Laden Patch for Endothelialized Tissue Engineering Applications

**DOI:** 10.3390/jfb12030045

**Published:** 2021-08-11

**Authors:** Yasaman Delkash, Maxence Gouin, Tanguy Rimbeault, Fatemeh Mohabatpour, Petros Papagerakis, Sean Maw, Xiongbiao Chen

**Affiliations:** 1 Division of Biomedical Engineering, University of Saskatchewan, 57 Campus Drive, Saskatoon, SK S7N 5A9, Canada; maxence.gouin@2020.icam.fr (M.G.); tanguy.rimbeault@2020.icam.fr (T.R.); fatemeh.mohabatpour@usask.ca (F.M.); petros.papagerakis@usask.ca (P.P.); 2 School of Engineering, Icam Site de Paris-Sénart, 34 Points de Vue, 77127 Lieusaint, France; 3 School of Engineering, Icam Site de Vendée, 28 Boulevard d’Angleterre, 85000 La Roche-sur-Yon, France; 4 College of Dentistry, University of Saskatchewan, 105 Wiggins Road, Saskatoon, SK S7N 5E4, Canada; 5 Graham School of Professional Development, 57 Campus Drive, Saskatoon, SK S7N 5A9, Canada; sean.maw@usask.ca

**Keywords:** 3D bioprinting, eggwhite-based bioink, albumin, vascularization

## Abstract

Three-dimensional (3D) bioprinting is an emerging fabrication technique to create 3D constructs with living cells. Notably, bioprinting bioinks are limited due to the mechanical weakness of natural biomaterials and the low bioactivity of synthetic peers. This paper presents the development of a natural bioink from chicken eggwhite and sodium alginate for bioprinting cell-laden patches to be used in endothelialized tissue engineering applications. Eggwhite was utilized for enhanced biological properties, while sodium alginate was used to improve bioink printability. The rheological properties of bioinks with varying amounts of sodium alginate were examined with the results illustrating that 2.0–3.0% (*w*/*v*) sodium alginate was suitable for printing patch constructs. The printed patches were then characterized mechanically and biologically, and the results showed that the printed patches exhibited elastic moduli close to that of natural heart tissue (20–27 kPa) and more than 94% of the vascular endothelial cells survived in the examination period of one week post 3D bioprinting. Our research also illustrated the printed patches appropriate water uptake ability (>1800%).

## 1. Introduction

Tissue engineering can be a way to promote tissue repair and regeneration. Fabricating living constructs by three-dimensional (3D) bioprinting has attracted considerable attention because this method offers more precise control over creating complex constructs or patches [1,2,3]. One of the most common 3D bioprinting methods is based on the extrusion principle, by which the biomaterial solution is mechanically driven through and then out of a nozzle (or printing head) to create 3D constructs layer by layer. This method has compatibility with a wide range of biomaterials, natural and synthetic [2,4,5,6]. Moreover, cells can be included in printing biomaterials (such mixtures are called bioinks) and located even in the innermost parts of the 3D construct [2,4,5,7,8,9,10]. For printing such constructs, the biomaterials should have appropriate mechanical properties and beneficial biological cues to support and facilitate cell functions such as attachment, growth, and proliferation. Naturally derived materials mainly present these biological cues, but often suffer from weak mechanical properties. On the other hand, synthetic materials are able to provide adjustable mechanical properties [11], typically have poor bioactivity, and often have to be functionalized by bioactive agents [4,5,11,12].

Albumin is a highly bioactive, easily acquired natural protein. This material has a low production cost versus many other proteins, such as collagen or fibronectin [13]. From the biological perspective, albumin as a coating layer in cell cultures has shown better efficiency than collagen and fibronectin as a mediator in enhancing cell-material attachment [14]. Albumin is biodegradable in the body environment, and its monomers (amino acids) are biocompatible [15]. It also has suitable mechanical properties such as good elasticity [16]. Albumin-derived electrospun patches have shown higher flexibility versus similar patches made of PLLA/PLGA and PCL [17]. Additionally, albumin is a competent drug carrier. It consists of helical turns of amino acids with multiple ligand binding sites, providing a high affinity to many drugs and growth factors for bonding and transporting [18,19]. This function is also beneficial for the implanted tissue-like construct in order to absorb cytokines and growth factors from the environment and release them gradually during the tissue remodeling [13,20]. Thus, the chances of integrating the implanted tissue patch with the surrounding healthy tissues improve. Albumin-based materials have been used in medicine and tissue engineering research in various areas such as skin [13], bone [21], lung [14], and heart [22]. For example, albumin-derived surgical sealant as a hemostatic adjunct for cardiac and vascular surgeries has shown excellent expansion and minimal inflammation within three months in pigs [23,24]. In wound healing applications, albumin in sponge form has shown higher angiogenesis than the collagen-derived sponges, a commonly used biomaterial in skin repair and tissue engineering [13,25]. This enhancement has been attributed to the significant adsorption of proangiogenic vascular endothelial growth factor (VEGF) by the applied eggwhite patches. Albumin can be derived from different sources. It is the most abundant protein in the blood plasma (approximately half of the total protein in the plasma) [16]. Although having this choice as an autogenic source of albumin is valuable, in the clinical setting, a significant amount of blood is required to harvest enough albumin, which makes the procedure invasive [13].

Alternatively, chicken eggwhite is an accessible, low-cost source of albumin. Eggwhite proteins are mainly ovalbumin, conalbumin, and lysozyme, which play essential biological roles, including embryo protection and development [26]. Biodegradation of these proteins down to their building blocks (amino acids) within the engineered tissue patch can provide the required nutrients for cells [22]. Accordingly, eggwhite constructs have been suggested as an available and suitable model for 3D cell culture studies [27], providing an appropriate mechanical and biological environment for cell proliferation and progression [28].

Notably, 3D printing of eggwhite is challenging due to its flow behavior, which can make it inappropriate for printing and forming 3D structures [29]. For improvement, it has been reported that eggwhite could be gelled using NaOH before printing to gain a tractable texture for 3D printing [30]. However, the addition of NaOH becomes an issue in tissue bioprinting as cells can be negatively affected in terms of their viability and functions, such as proliferation, morphology, and cytoskeletal distribution [31]. The aim of the present study was to develop a printable bioink based on chicken eggwhite with sodium alginate being an alternative cationic material crosslinkable with CaCl_2_ in order to bioprint cell-laden patches. Another objective was to characterize such patches in vitro for potential use in endothelialized tissue engineering. After rheological characterization and test printing of various eggwhite-sodium alginate (EW-Alg) solutions containing 1.0% to 3.0% (*w/v*) sodium alginate, printable solutions were mechanically characterized to identify the optimum printed construct for biological characterization.

## 2. Materials and Methods

### 2.1. Preparation and 3D Printing of EW-Alg 3D Constructs for Mechanical Characterization

Alginate powder (medium viscosity alginate, sodium salt from brown algae, Sigma-Aldrich) was dissolved directly in the intact pasteurized eggwhite (12 g protein per 100 mL, Naturegg Simply Egg Whites) in different percentages of 1.0, 1.5, 2.0, 2.5, and 3.0% (*w*/*v*) and was stirred using a magnetic stirrer at room temperature (25 °C) for 3 h. Solutions were neutralized by 0.5 mM HCL to pH ~7 and then were centrifuged to reduce the bubbles formed by the mixing process of alginate and eggwhite. Patches were fabricated at room temperature by a pneumatically controlled 3D Bioplotter (EnvisionTEC GmbH, Gladbeck, Germany). The structure of the patches was designed using Magics EnvisionTEC (V13, Materialise), Bioplotter RP (V2.9, EnvisionTEC GmbH), and VisualMachine BP (V2.2; EnvisionTEC GmbH). Patches were printed in 10 layers with a surface area of 12 × 12 mm^2^ and a height of 8 mm. The inner strand structure was designed to have a 1.5 mm distance between strands with a 90° hatch pattern. Plastic dispenser tips (25-gauge, EFD Nordson, East Providence, RI, USA) were used for manufacturing all of the patch groups. Speeds of 9, 10, and 11 mm/s and pressures of 0.3, 0.5, and 0.7 bar were used to print EW-2.0%Alg, EW-2.5%Alg, and EW-3.0%Alg constructs, respectively. Strands were dispensed into a crosslinker bath that consisted of 25 mM CaCl_2_ (Sigma-Aldrich, St. Louis, MO, USA) and 0.25% (*w*/*v*) polyethyleneimine (PEI) (MW 60,000, 50% *w*/*w* in H2O, Alfa Aesar). Sample dishes had been coated with 0.1% (*w*/*v*) PEI aqueous solution in the incubator environment one day before printing to enhance the surface adhesion to the ink [32]. PEI solution was replaced with the crosslinker bath right before printing. Printed patches were kept in 500 mM CaCl_2_ overnight. Then samples were washed five times in water and kept in PBS for mechanical characterizations.

### 2.2. Rheological Characterization

To study the flow behavior of each printing blend, rheological measurements were performed at two temperatures (25 and 37 °C) using a rheometer (RVDV-III Brookfield, Stoughton, MA, USA) with a cone and plate geometry of 40 mm diameter and 2° angle. For each EW-Alg blend, 2 mL of EW-Alg was initially placed inside the rheometer plate and set at the desired temperature (25 or 37 °C). After reaching the steady-state temperature, rotation started with speed increments of 5.0, 1.0, 0.5, and 0.1 rpm for the blends of EW-1.5%Alg, EW-2.0%Alg, EW-2.5%Alg, and EW-3.0%Alg, respectively. Shear rate, shear stress, and viscosity were sampled using Brookfield software. Shear stress versus shear rate graphs and viscosity versus shear stress graphs were then plotted based on the collected data for each ink. For each concentration, at least five tests were run per temperature point.

### 2.3. Swelling and Degradation Behavior

To measure the 3D printed patches’ ability to uptake a body-like fluid, fabricated patches were dehydrated using a freeze dryer (Labconco, Kansas City, MO, USA) overnight. All the dried patches were weighed (W_0_) and then immersed in PBS solution and kept inside the incubator at 37 °C, 5% CO_2_. After 24 h, patches were taken out from the solution and weighed (W_W_) after the excess solution on the surface was removed. Results were calculated according to Equation (1) and the mean value of quadruplicate measurements for each group was presented.

% Swelling = (W_W_ − W_0_)/W_0_ × 100 (1)

To analyze the printed patches’ biodegradation behavior in a body-like environment, the lyophilized printed patches were immersed in PBS and placed inside the incubator for 28 days. PBS was changed twice a week to keep the solution fresh with constant ion concentrations. Every week, four samples from each group were taken out to monitor the degradation profile.

### 2.4. Mechanical Strength

To examine the mechanical strength of patches under a compressive force, a compression test was performed using a Bose biodynamic mechanical testing machine (BioDynamic 5100 Bose, USA). For evaluating the compressive elastic modulus of the patches, unconfined compression with a preload of 1 N and a total displacement of 5 mm at a rate of 0.01 mm/s was applied to each patch at room temperature. Compressive elastic moduli of patches were obtained from the related stress–strain curve slopes in the elastic deformation region. Five patch samples from each blend of EW-Alg were tested. The average values for each set of tests were calculated and presented.

### 2.5. Cell Culture

Human umbilical vein endothelial cells (HUVECs) (ATCC, Rockville, MD, USA) were cultured in a complete culture medium made of Dulbecco’s modified Eagle’s medium (DMEM, Gibco), 10% hypoxanthine-aminopterin-thymidine (HAT, Gibco), 10% fetal bovine serum (FBS, Gibco), and 1% penicillin-streptomycin antibiotics (PS, Sigma-Aldrich) [33]. After the cells covered the culture flask (80% confluency), cell subculture was performed. Cells were detached using 0.025% trypsin treatment for 2 min. Trypsin then was neutralized by the complete culture medium (containing FBS). The dissociated cell suspension was centrifuged at 1200 rpm at 4 °C for 5 min. The supernatant was removed, and the pellet was cultured again. Passage IV cells were used for 3D bioprinting.

### 2.6. Bioprinting the Cell-Laden Patches

**Figure 1:**
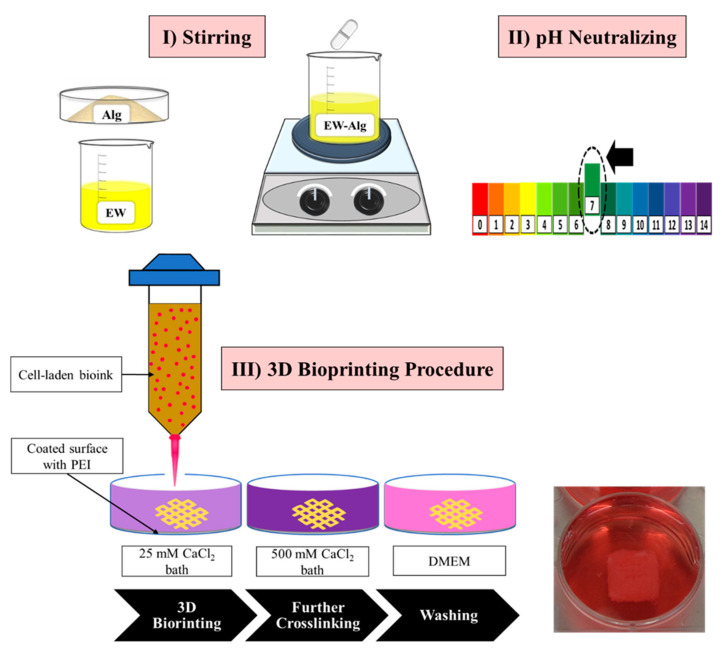
A summary of the HUVEC-laden patch 3D bioprinting procedure.

To prepare the cell-laden bioink, alginate powder was sterilized under a UV lamp (250 nm wavelength) for 2 h and then was mixed with pasteurized eggwhite to prepare the EW-2.0%Alg blend. EW-Alg blend then was neutralized to pH ~7 by micro-filtered HCL. The cultured HUVECs’ suspension with a density of 1.25 × 10^6^ cells per milliliter was added to the EW-Alg blend at a ratio of 1 to 2 and was then stirred gently to achieve a homogenous bioink. All the printing plates were coated with autoclaved 0.1% (*w*/*v*) PEI one day before printing. The cell-laden bioink was then loaded into the bioplotter dispenser and was 3D bioprinted into the autoclaved 25 mM CaCl_2_ bath containing 0.25% PEI. Shortly after printing, patches were transferred into the 500 mM CaCl_2_ bath for 15 min and were subsequently washed with the complete cell culture medium three times (see Figure 1).

### 2.7. Cell Viability Assay

Calcein AM and propidium iodide (PI) (AnaSpec, Fremont, CA, USA) were dissolved in PBS with a final concentration of 1.0 and 0.5 μL/mL for staining the live and dead cells, respectively. At each time point, after adding the dye solution to the printed patches, samples were incubated for 30 min. A fluorescent microscope (EVOS M5000) was used for imaging. Staining and imaging were performed on days 1, 4, and 7 after printing. For each time point, three patches were prepared. Eight images were taken and analyzed per day using Image J software, and the cell viability was obtained using Equation (2):

% Cell Viability = (Live cells)/(Live cells + Dead cells) × 100(2)

### 2.8. Statistical Analysis

The statistical significance of the result was calculated using a one-way analysis of variance (ANOVA). Pairwise comparisons were performed using the *T*-test in Excel 2016. Results were considered statistically significant for *p*-values < 0.05.

## 3. Results

### 3.1. Rheological Characterization

**Figure 2:**
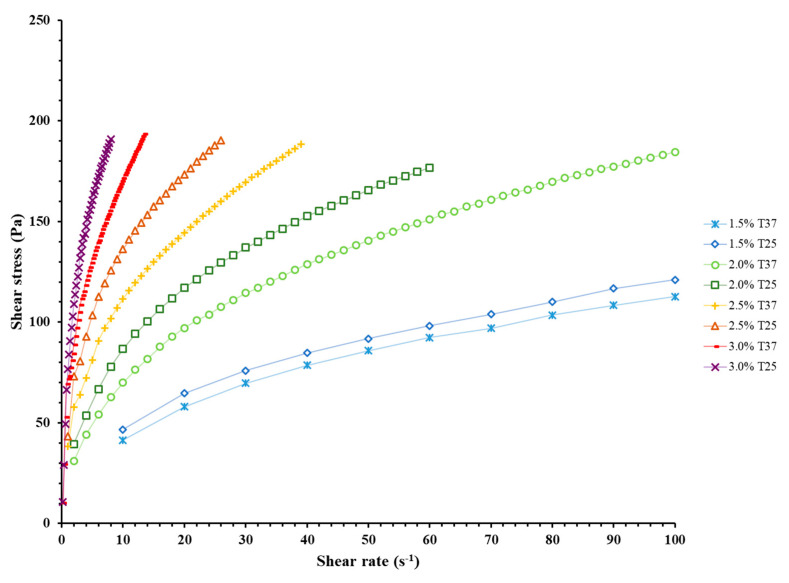
Rheological curves of shear stress versus shear rate for all prepared EW-Alg blends in temperatures of 25 and 37 °C. In order to show all the charts in one graph, the shear rate axis was truncated at 100 s^−1^.

**Figure 3:**
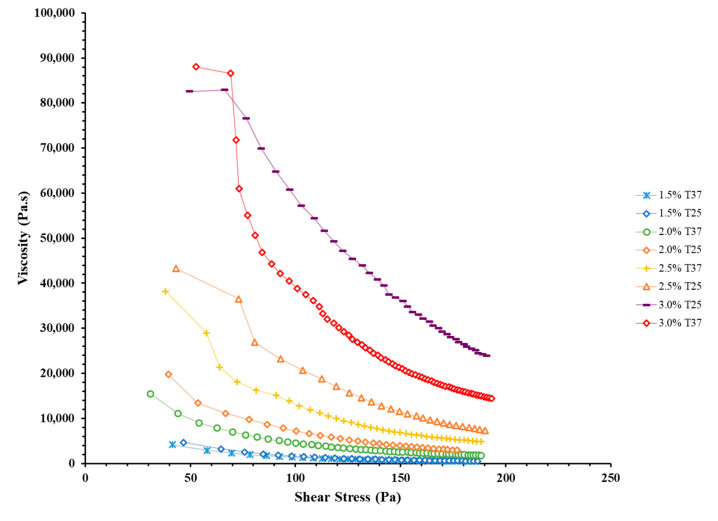
Rheological curves of viscosity versus shear stress for all prepared EW-Alg blends in temperatures of 25 and 37 °C.

The rheological properties of the ink are critical to sustaining successful 3D printing [34]. Alginate concentration and blend temperature were studied as independent variables in the rheological studies of the EW-Alg inks. Results showed that the ink significantly gains a thicker texture and higher viscosity per each additional 0.5% alginate (see Figure 2 and Figure 3). In addition, all the EW-Alg blends showed a non-Newtonian shear-thinning behavior in which the viscosity decreases with the increase of shear stress.

Furthermore, the temperature had an inverse correlation with viscosity in each group of inks. All inks at 25 °C possessed thicker textures as compared to their 37 °C states. However, the impact of temperature in this observed range (25 to 37 °C) is not as high as the impact of alginate concentration in ink.

### 3.2. Water Uptake Behavior and Biodegradability

**Figure 4:**
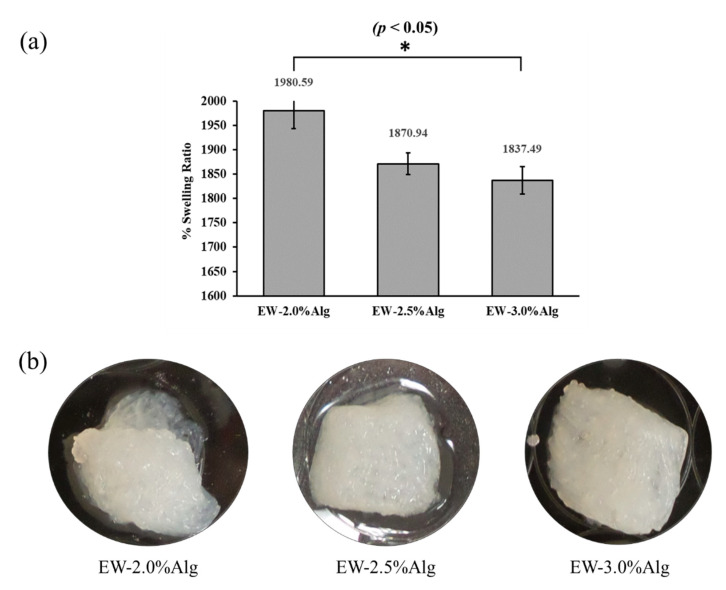
(**a**) Swelling behavior of 3D printed EW-Alg patches after 24 h in PBS solution; (**b**) Degraded patches after 28 days in PBS.

All three groups of patches showed swelling ratios of more than 1800%. The highest value was exhibited by the EW-2.0%Alg patch, representing a strong ability to uptake the PBS as a liquid representative of body fluids. The highly porous structure of the printed patches can deliver and retain the body fluid in the damaged area of the tissue and provide an enriched environment that can expedite vascularization and tissue regeneration (see Figure 4a and Appendix A).

In the biodegradation test, no significant weight changes were observed in the samples on days 7, 14, and 21. However, a decline in mechanical properties was tangible after day 21. On day 28, surface strands were dissociating, and constructs were very fragile to the touch (see Figure 4b).

### 3.3. Mechanical Strength

**Figure 5:**
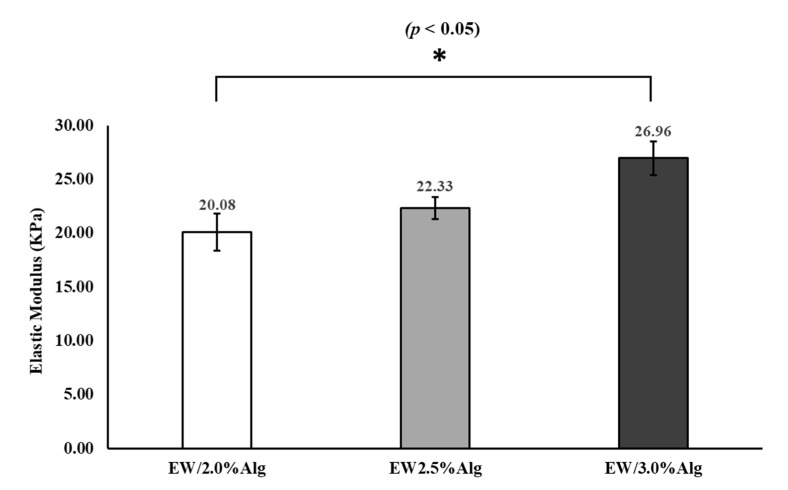
Compressive elastic moduli of 3D printed EW-Alg patches.

As illustrated in Figure 5, by increasing the alginate concentration, the elastic modulus increased. This increase in elastic modulus reflected the crosslinked structures within the construct due to having a more ionically condensed blend in higher concentrations. i.e., due to having more sodium alginate in the constructs, the proportion of Na^+^ exchanged with Ca^2+^ became greater, and more crosslinking formed more robust constructs. ANOVA analysis showed a *p*-value of <0.05 between all groups. Pairwise T-test comparisons showed a significant difference in elastic moduli between EW-2.0%Alg and EW-3.0%Alg samples, although the change was not very notable per each additional 0.5% of alginate.

### 3.4. Cell Viability

**Figure 6:**
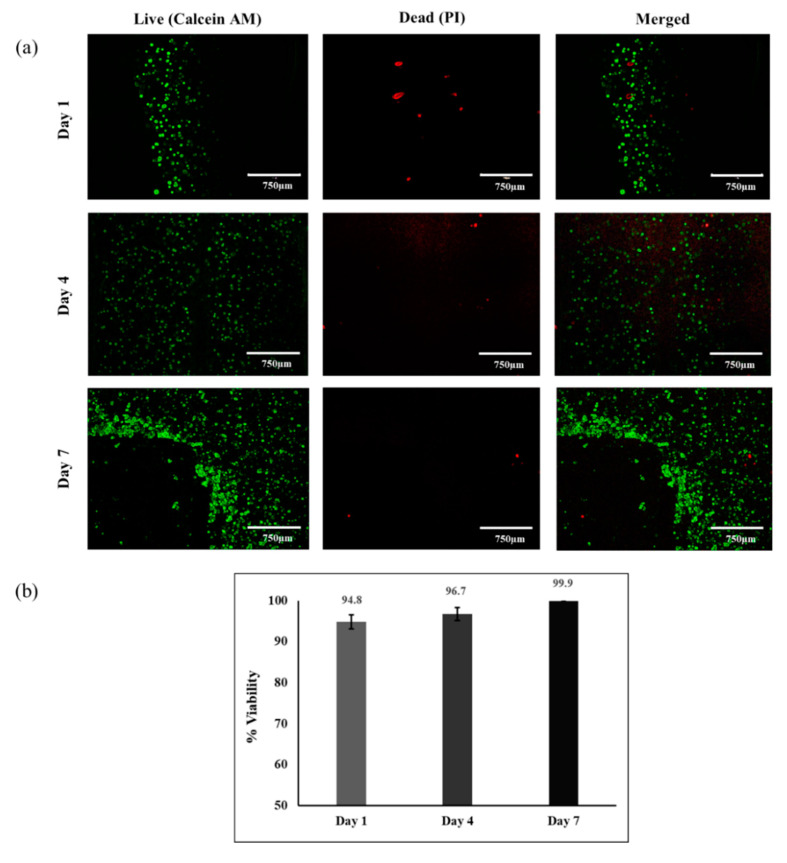
Cell viability and proliferation of 3D bioprinted HUVECs within the EW-2.0%Alg bioink by live/dead assay 1, 4, and 7 days after printing. (**a**) Fluorescent microscopy; (**b**) % cell viability.

The live/dead assay was conducted on days 1, 4, and 7 after printing. Results in Figure 6 show that HUVECs maintained their viability within the 3D printed patch, and the ratio of dead cells to live cells remained very low (<6%) at all time points after bioprinting. Cell multiplication shows that the high rate of HUVEC proliferation led to a dense cellular patch on day 7. These results indicate that the optimum EW-based bioink (EW-2.0%Alg) could support cell viability during the printing process as well as cell proliferation one week post-printing.

## 4. Discussion

Three-dimensional bioprinting, as an additive manufacturing method, has become a popular approach in fabricating tissue patches for various tissue types. However, material selection has been challenging as the natural materials cannot present mechanical properties strong enough to match the organic tissue. On the other hand, synthetic materials lack beneficial bioactivity. In this study, as a source of albumin protein with ECM-like mechanical properties and excellent biological properties, eggwhite was used as the main part of the bioink to 3D bioprint a cell-laden patch with vascular endothelial cells.

As the intact eggwhite fluid lacks extrudability, alginate as an extrusion enhancer was added to the plain eggwhite in the minimum possible amount. As a result, eggwhite could benefit from the ionic crosslinking feature of alginate to form a stable configuration in the presence of CaCl_2_ after 3D bioprinting.

Rheological characterization showed that the addition of alginate resulted in viscous, shear-thinning bioinks with desirable flow properties. According to rheological studies, extruding inks with higher alginate concentrations requires higher printing pressures. Higher printing pressures means more shear stress applied to the loaded cells within a bioink. Based on the type of cells in question, high shear stresses can be harmful in different ways, such as damaging the cell membrane and/or changing cell behavior and cell fate in the long term [35].

Blends of EW-Alg were prepared with 1.0 to 3.0% (*w*/*v*) alginate in eggwhite. However, blends of EW-1.0%Alg and EW-1.5%Alg were too watery to print and could not be structured as reproducible patches (Appendix B).

In tissue fibrosis, microvascular damage can be observed at different scales. This issue can slow down or prevent further repair of the tissue [36]. Therefore, after implanting an engineered cell-laden patch, due to the distance of cells from the vascular system (especially in the internal parts of a thick implant), the transfer of nutrients and waste materials to and from the transplanted cells is a major concern [37]. In this case, the albumin, considering its drug delivery properties, has the potential to absorb the soluble growth factors from the environment and offer them to the cell-containing implant. Thus, the albumin can stimulate a localized vascular system. Swelling measurements of the 3D printed EW-Alg patches in the present study demonstrated the strong ability of eggwhite to uptake the ionic fluid with a slightly higher amount of swelling for EW-2.0%Alg among the observed groups.

Following the swelling test, all the constructs showed signs of biodegradation within a month. This is a positive feature for an engineered tissue-like implant as wound healing typically shows significant progress within approximately 28 days of injury, when cell proliferation overlaps the beginning of tissue remodeling. This time is reported as 10–14 days for cutaneous wound healing, 14–28 days for ligament repair [38], and 14–35 days for bone remodeling [39]. Three-dimensional patches tested for biodegradability here were cell-free constructs. We speculate that the degradation rate might have been higher for the cell-laden patch as the cells can digest the patch during their proliferation. Another influential factor in degradation is fluid flow (such as blood flow). The performed degradation test in this study was conducted statically, whereas patches may degrade faster in the presence of body fluid circulation [40]. In addition, tissue movements (such as heartbeats) may also speed up this degradation rate.

The compressive elastic moduli of the EW-Alg 3D printed patches (20–27 kPa) are close to those of the porcine heart tissues reported in the literature. Porcine cadaver heart (LV part) and its decellularized form (heart ECM) have shown compressive elastic moduli in the ranges of ~2.0 to 8.5 and ~1.5 to 6.0 kPa, respectively [41]. Moreover, the human cadaver limb compressive elastic modulus is in the range of ~20 to 38 kPa for males and ~10 to 32 kPa for females [42]. On the other hand, many commercial silicon-based materials used in soft tissue modeling have compressive elastic moduli close to the values of the fabricated EW-Alg patches. Dragon Skin (Smooth-On, Easton, PA, USA) at 20–850 kPa and Semicosil 921 (Wacker Solutions, Adrian, MI, USA) at 25 kPa [43] are just two of these products. Therefore, fabricated EW-Alg patches may have promise as a platform in stimulating soft tissues.

**Figure 7:**
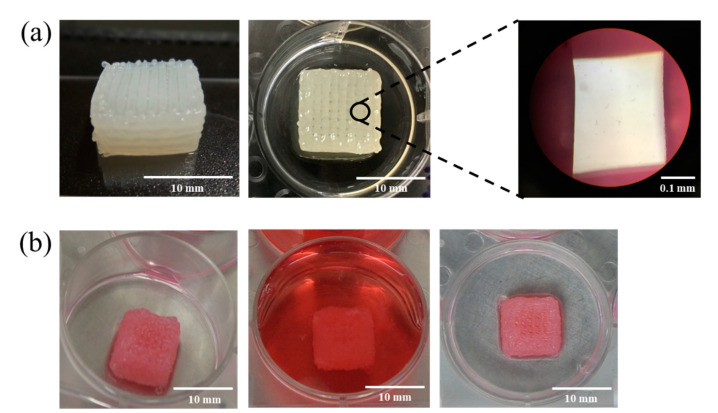
(**a**) Macroscopic and microscopic views of 3D printed strands with uniform texture and configuration; (**b**) 3D bioprinted HUVEC-laden patches (12 layers) from different views.

As with the cell-free patches, cell-laden EW-2.0%Alg bioink could be 3D bioprinted up to 12 layers, with high fidelity (see Figure 7). To benefit the most from EW-Alg bioink, efforts were made to minimize parameters affecting cell viability by using the bioink with the lowest viscosity and by printing with a wide gauge needle dispenser (25-gauge) to decrease the applicable shear stress.

Previous studies have shown that eggwhite is a biocompatible, non-toxic biomaterial [14] and that alginate is a commonly used biomaterial in cell printing [44]. Our present study examined the HUVEC viability in the EW-2%Alg printed construct post-bioprinting, which could be affected by the mechanical forces that the cells experienced during the bioprinting process. For the examination, we only used the live/dead staining assay in the present study. For the application of this printed cell-laden patch in endothelial tissue regeneration, further characterizations are recommended, including studies to examine the printed cell morphology and activity within the patch.

Alginate hydrogel has weak mechanical properties [45], especially for hard tissue applications, and low bioactivity in cell adhesion [46]. For strengthening the construct, alginate has been mostly used along with mechanically stronger materials such as ceramics (e.g., hydroxyapatite [47]) or synthetic biomaterials (e.g., PCL [48]). On the other hand, to improve cell adhesivity, functionalizing the alginate-based patches with cell-adhesive agents such as the Arg-Gly-Asp (RGD) sequence is common in biofabrication research [46,49]. In the present study, neither synthetic biomaterial nor RGD modification of alginate was utilized since the eggwhite could address the alginate’s mechanical and biological shortcomings, resulting in a stronger natural material with higher bioactivity than simple alginate.

**Figure 8:**
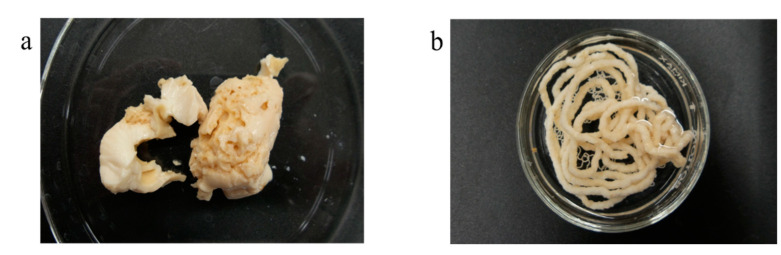
Cooked form of EW-2.0%Alg after autoclaving. (**a**) Porous bulk; (**b**) extruded form.

We also observed that cooking the EW-Alg blends converted the blends into different porous structures which can also be extruded and 3D printed. However, in this case, where the results were solid gels, incorporating the cells for 3D cell-laden bioprinting becomes almost impossible. The alternative approach is culturing the cells on a cooked 3D printed scaffold. Cooking the scaffold in addition to creating a microporous structure within each strand can also be an excellent method of sterilization. Figure 8 shows some attempts at creating such designs.

## 5. Conclusions

This paper presents our study on the development of eggwhite-based hydrogels for bioprinting cell-laden patches for vascularized tissues such as cardiac tissue. We showed that a small amount of sodium alginate can be used along with EW to improve the printability and mechanical strengths of 3D printed cell-laden patches while having no significant negative effects on cell viability. The mechanical characterization of the printed patches showed their high swelling ratio, suitable biodegradability in the simulated body environment, and mechanical strength similar to that of natural muscle tissue. The decent biocompatibility of the bioink was observed as >94% of the HUVECs loaded within the EW-Alg bioink were viable (with the highest amount of eggwhite) after bioprinting. The bioprinted cell-laden patch with its vascularization potential seems promising for future in vivo studies.

## Figures and Tables

**Figure A1:**
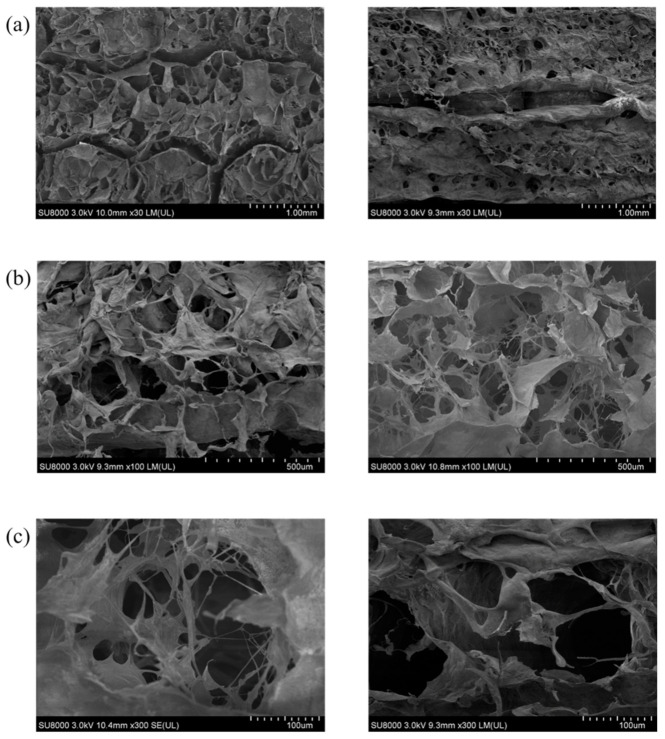
SEM images showing porous structure of strands in EW-2%Alg construct. (**a**) 30× magnification; (**b**) 100× magnification; (**c**) 300× magnification.

**Figure A2:**
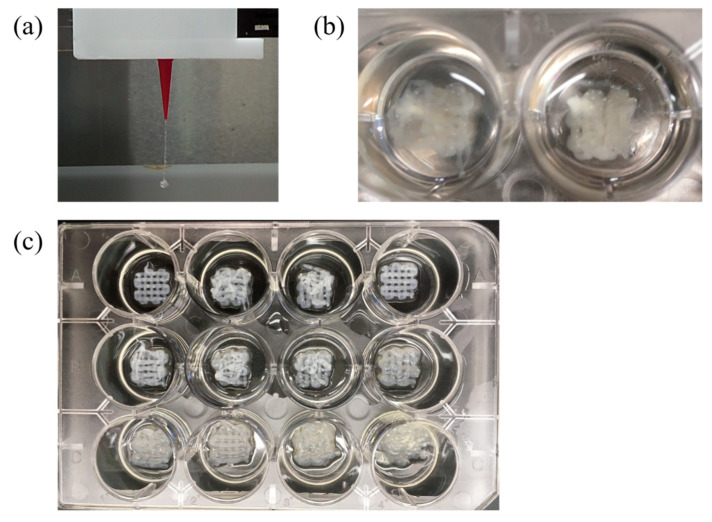
Difficulties during the 3D printing procedure of EW-1.5%Alg blend. (**a**) Unattached strands leading to clogging of the needle; (**b**) instability of the printed structure in the crosslinker bath; (**c**) low fidelity and resolution in printing multi-layer constructs.

## Data Availability

The datasets used and/or analyzed during the current study are available from the corresponding author on reasonable request.
